# EFFICACY OF ULTRASOUND-GUIDED INTRA-ARTICULAR HYALURONIC ACID INJECTION IN THE MANAGEMENT OF ADHESIVE CAPSULITIS: A RANDOMIZED CONTROLLED TRIAL

**DOI:** 10.2340/jrm.v58.44901

**Published:** 2026-05-14

**Authors:** Chih-Ya CHANG, Ching-Ting CHEN, Yung-Hsiang LIN, Chi-Fu CHIANG, Yi-Wen MAO, Li-Wei CHOU

**Affiliations:** 1 Department of Physical Medicine and Rehabilitation, College of Medicine, National Defense Medical University, Tri-Service General Hospital, Taipei; 2 Department of Physical Therapy and Assistive Technology, National Yang Ming Chiao Tung University, Taipei; 3 Maxigen Biotech Inc, Taoyuan; 4 Research & Design Center, TCI Co Ltd, Taipei, Taiwan

**Keywords:** adhesive capsulitis, frozen shoulder, hyaluronic acid, intra-articular injection, ultrasound-guided procedure, physical therapy, shoulder pain

## Abstract

**Objective:**

To compare the efficacy and safety of ultrasound-guided intra-articular hyaluronic acid injection versus supervised rehabilitation in patients with frozen-phase adhesive capsulitis.

**Design:**

Single-center, parallel-group, randomized controlled trial with 26-week follow-up.

**Subjects/Patients:**

Forty-six adults aged 40–70 years with clinically and radiologically confirmed frozen-phase adhesive capsulitis.

**Methods:**

Participants were randomly assigned to receive either three weekly ultrasound-guided intra-articular hyaluronic acid injections or supervised physical therapy twice weekly for six weeks. Outcomes assessed at baseline and at weeks 4, 6, 8, and 26 included the Shoulder Pain and Disability Index (SPADI) and shoulder range of motion.

**Results:**

Both groups achieved significant within-group improvements in SPADI and range of motion over 26 weeks (all *p* < 0.01 vs. baseline). No statistically significant between-group differences were observed in total SPADI or its pain and disability subscales at any follow-up time point (all *p* > 0.05). At week 26, the hyaluronic acid group’s mean total SPADI decreased from 43.0 **±** 17.7 to 16.1 **±** 12.8 (*p* < 0.01), while the rehabilitation group improved from 52.0 **±** 18.4 to 23.2 **±** 21.8 (*p* < 0.01). Pain subscores were reduced by 64.3% (hyaluronic acid) and 56.1% (rehabilitation); function subscores decreased by 60.9% and 54.8%, respectively (all *p* < 0.01). Both groups showed significant gains in active and passive flexion, abduction, and external rotation, without significant between-group differences (all *p* > 0.05). No treatment-related adverse events were observed.

**Conclusion:**

Ultrasound-guided hyaluronic acid injection and supervised rehabilitation produced comparable and clinically meaningful improvements in pain, function, and shoulder mobility in frozen-phase adhesive capsulitis.

## LAY ABSTRACT

Frozen shoulder, also known as adhesive capsulitis, causes long-lasting shoulder pain and stiffness that make daily tasks difficult. This study compared 2 common treatments during the stiff or “frozen” stage: hyaluronic acid injections given into the shoulder joint under ultrasound guidance, and supervised physical therapy. Forty-six patients were followed for 6 months. Both treat-ments improved shoulder movement, reduc-ed pain, and restored function. By the end of the study, pain and functional limitation had improved by more than 50% in both groups. Improvements were similar between treatments, and no side effects occurred. These findings suggest that ultrasound-guided hyaluronic acid injection is a safe and effective non-steroidal alternative for patients with frozen shoulder, especially for those who cannot receive corticosteroids or prefer fewer therapy sessions.

Adhesive capsulitis (AC), commonly referred to as frozen shoulder, is a progressive musculoskeletal disorder characterized by chronic shoulder pain and marked limitation of both active and passive range of motion (ROM). Affecting approximately 2–5% of the general population, it predominantly occurs in individuals aged 40 to 65 and shows a higher prevalence among women and those with comorbidities such as diabetes mellitus or thyroid dysfunction (1, 2). Although AC is often self-limiting, its natural course can extend from 1 to 3 years, resulting in prolonged functional impairment and diminished quality of life (1).

The clinical course of AC typically progresses through 3 overlapping phases: the painful (freezing) phase, the stiffness-dominant (frozen) phase, and the resolution (thawing) phase (3). The frozen phase represents a key therapeutic window, during which inflammatory processes subside and mechanical capsular contracture becomes the predominant cause of motion restriction (3, 4).

Diagnosis of primary adhesive capsulitis is based on clinical criteria, including shoulder pain lasting for at least 3 months, significant restriction of active and passive ROM (≥ 30% compared with the contralateral side) in at least 2 planes of motion, and exclusion of other intra-articular shoulder disorders through imaging. A history of trauma or prior shoulder surgery is typically absent, supporting the diagnosis (5).

Pathophysiologically, AC involves synovial inflammation and capsular fibrosis driven by upregulated fibrogenic cytokines such as transforming growth factor-beta (TGF-β) and interleukin-6 (IL-6), which promote fibroblast proliferation and excessive extracellular matrix deposition (6). Magnetic resonance imaging (MRI) and ultrasonography can demonstrate characteristic capsular thickening and synovitis, but the diagnosis remains primarily clinical (7).

Treatment strategies for AC include nonsteroidal anti-inflammatory drugs (NSAIDs), physical therapy, corticosteroid injections, and, in refractory cases, procedures such as manipulation under anesthesia or arthroscopic capsular release (8). A recent network meta-analysis further compared pharmacological interventions and highlighted the growing role of HA as a non-steroidal alternative with favorable safety and efficacy profiles (9). Among conservative treatments, physical therapy remains the cornerstone, focusing on restoring ROM and shoulder mechanics (5). Corticosteroid injections are commonly used but may offer only short-term relief and carry risks of tendon weakening with repeated use (10).

Hyaluronic acid (HA), a high-molecular-weight polysaccharide naturally present in synovial fluid, has emerged as an alternative therapeutic option. Its viscoelastic and anti-inflammatory properties may help improve joint lubrication, modulate synovitis, and reduce pain (11). Several randomized controlled trials have investigated the efficacy of intra-articular HA injections in AC, with some showing benefits in pain relief and functional recovery comparable to corticosteroids or physical therapy (12–14). However, findings across studies remain inconsistent, highlighting the need for further well-designed trials focusing specifically on the frozen phase of the disease (12, 15). Recent meta-analyses have also underscored the limited evidence regarding the comparative effectiveness of pharmacological agents during this stage, reinforcing the importance of targeted investigations such as the present study (9).

Therefore, the present randomized controlled trial aimed to evaluate the clinical efficacy and safety of ultrasound-guided intra-articular HA injections compared with structured physical rehabilitation in patients with frozen-phase adhesive capsulitis, over a 26-week follow-up period.

## MATERIALS AND METHODS

### Study design and ethical considerations

**Fig. 1:**
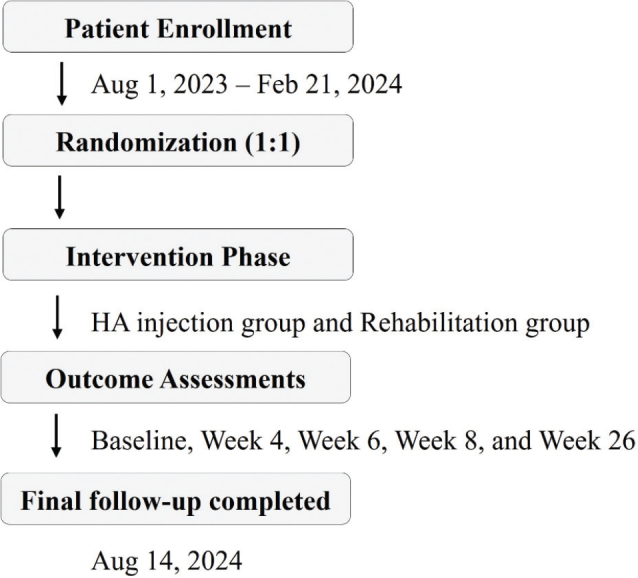
Graphical timeline of patient enrollment, randomization, intervention, and follow-up assessments.

This was a single-center, parallel-group, 2-arm randomized controlled trial conducted at Tri-Service General Hospital (Taipei, Taiwan). Patient enrollment was conducted between 1 August 2023 and 21 February 2024, and the final follow-up assessment was completed on 14 August 2024. The study timeline is illustrated in Fig. 1. The study protocol was approved by the institutional review board (IRB approval number: C202205161) and conducted in accordance with the Declaration of Helsinki. Written informed consent was obtained from all participants prior to enrollment. The trial was retrospectively registered at ClinicalTrials.gov (NCT05983081) on 9 August 2023, shortly after the initiation of patient enrollment on 1 August 2023. The study protocol, including predefined primary and secondary outcomes and the statistical analysis plan, had been approved by the institutional review board prior to recruitment, and no changes were made after trial commencement. The trial was reported in accordance with CONSORT 2010 guidelines (16), and no deviations from the original protocol occurred.

### Participants

Eligible participants were recruited from outpatient clinics and diagnosed with frozen-phase primary adhesive capsulitis by orthopedic specialists. Diagnosis was based on clinical history, physical examination, and imaging studies, including plain radiography and either musculoskeletal ultrasound or, when clinically indicated, magnetic resonance imaging (MRI), to exclude differential diagnoses such as full-thickness rotator cuff tears or calcific tendinopathy (7). Frozen-phase AC was defined according to commonly accepted clinical criteria and imaging findings (3, 4), characterized by predominant stiffness lasting at least 3 months, minimal resting pain (Visual Analog Scale [VAS] < 3), resolution of nocturnal pain, and restricted shoulder mobility.

While external rotation restriction is often considered the hallmark of adhesive capsulitis (17), abduction was also included based on its relevance to daily function and consistent involvement in the frozen phase (4, 6). The average duration of symptoms prior to enrollment was 13.46 ± 17.11 months in the HA group and 7.72 ± 8.08 months in the rehabilitation group (*p* = 0.165), and this information is summarized in Table I.

**Table I:** Baseline demographic and clinical characteristics of patients in the hyaluronic acid (HA) injection group and rehabilitation group

Characteristic	Group	HA (*n* = 21)	REH (*n* = 25)	*p*-value
Gender	Female (*n*)	14	14	
	Male (*n*)	7	11	
Age, years, mean ± SD		59.38 ± 7.5	59.42 ± 7.43	0.99
Height, cm, mean ± SD		161.67 ± 7.7	163.08 ± 7.22	0.52
Weight, kg, mean ± SD		60.76 ± 9.92	65.65 ± 12.78	0.16
BMI, kg/m^2^, mean ± SD		23.19 ± 2.99	24.58 ± 3.86	0.186
Symptom duration, months, mean ± SD		13.46 ± 17.11	7.72 ± 8.08	0.165
Diabetes mellitus (DM)	Yes (*n*)	6	5	0.547
	No (*n*)	15	19	
Hypertension (HTN)	Yes (*n*)	6	10	0.36
	No (*n*)	15	14	
Smoking	Yes (*n*)	1	3	0.363
	No (*n*)	20	21	
Alcohol	Yes (*n*)	3	2	0.56
	No (*n*)	18	21	
Treatment side	Left (*n*)	13	14	0.685
	Right (*n*)	8	11	

HA: HA injection group; REH: rehabilitation group.

Detailed inclusion and exclusion criteria are presented in Box 1.

### Randomization and interventions

Participants were randomized in a 1:1 ratio to either the HA injection group or the rehabilitation (REH) group using a computer-generated block randomization schedule to ensure balanced allocation. Random sequence was generated using a computer-based random number generator. Block randomization with a block size of 4 was used. The allocation sequence was concealed using sequentially numbered, sealed opaque envelopes. The random sequence was generated by a statistician not involved in patient enrollment; the study coordinator enrolled and assigned participants.

*HA injection group.* Participants received 1 ultrasound-guided intra-articular injection of sodium hyaluronate (Artibest^®^, 60 mg/3 mL, 2%; Maxigen Biotech Inc, Taiwan) per week for 3 consecutive weeks. All injections were performed via a posterior approach using a sterile technique and real-time ultrasound guidance to ensure accurate delivery into the glenohumeral joint. Ultrasound guidance has been shown to enhance injection precision and improve clinical outcomes in patients with adhesive capsulitis (18).

Box 1 Inclusion And Exclusion Criteria For Study Participation

**Table:** 

**Inclusion criteria:** Age 40–70 years Clinical diagnosis of frozen-phase primary adhesive capsulitis Persistent shoulder stiffness ≥ 3 months Minimal resting pain (VAS < 3) and absence of nocturnal pain ≥ 30% reduction in at least 2 planes (flexion, abduction, or external rotation) compared with the contralateral side, or ≥ 50% reduction in abduction alone Ability and willingness to comply with study procedures and follow-up visits **Exclusion criteria:** Imaging-confirmed full-thickness or massive rotator cuff tears Calcific tendinopathy Systemic inflammatory or autoimmune diseases (e.g., rheumatoid arthritis, systemic lupus erythematosus) Prior shoulder fracture or surgery on the affected side Intra-articular shoulder injection within the previous 3 months Neurological disorders (e.g., cervical radiculopathy) Pregnancy or lactation Active malignancy Medically unstable conditions (e.g., recent stroke, Parkinson’s disease) Cognitive impairment affecting protocol adherence

*Rehabilitation group.* Participants in the control group underwent supervised physical therapy twice weekly for 6 consecutive weeks. Each 30-min session included joint mobilization, passive and active-assisted stretching, and strengthening exercises targeting the rotator cuff and scapular stabilizers. Supervised physical therapy was provided according to a standardized rehabilitation program, with minor individualized adjustments made by licensed physical therapists based on patient tolerance and clinical judgment. The HA injection group did not receive any physical therapy, as the aim of this study was to evaluate the independent efficacy of HA compared with rehabilitation alone. Clinical assessments were performed at baseline and at weeks 4, 6, 8, and 26.

### Outcome measures

The primary outcome was the change in total Shoulder Pain and Disability Index (SPADI) score (overall composite of pain and function subscales) from baseline to week 26. The SPADI is a validated, self-administered questionnaire that assesses shoulder pain and functional limitation, with high reliability and responsiveness in adhesive capsulitis (19). Scores range from 0 to 100, with higher scores indicating greater pain and functional disability.

Secondary outcomes included measurements of active and passive shoulder range of motion (ROM) in flexion, abduction, and external rotation. Range of motion was assessed using a standard goniometer by a single blinded examiner to minimize inter-observer variability. Passive ROM was measured at the maximal attainable joint angle, defined as the point at which further movement was not possible due to mechanical restriction or patient tolerance. Other outcome measures, such as the SPADI questionnaire, were patient-reported and were not blinded. No changes were made to outcome measures after trial commencement. Internal rotation was not included among the measured ROM parameters due to its limited measurement reliability and high inter-subject variability in outpatient clinical settings. Flexion, abduction, and external rotation were selected based on their clinical relevance and consistent use in prior adhesive capsulitis trials.

### Statistical analysis

Sample size was estimated based on an expected between-group difference of 10 points in SPADI (SD = 12) from Mao et al. (12), with 80% power and two-tailed α = 0.05, requiring at least 21 participants per group.

Continuous variables are presented as mean ± SD, categorical variables as counts and percentages. Data normality was assessed using the Shapiro–Wilk test. Although minor deviations from normal distribution were observed in some variables, parametric tests were retained given the robustness of independent-sample *t*-tests and repeated-measures ANOVA in moderate sample sizes. Between-group comparisons used independent-sample *t*-tests, and within-group changes were evaluated by repeated-measures ANOVA. Statistical significance was set at *p* < 0.05.

All analyses were performed with SPSS v26.0 (IBM Corp, Armonk, NY, USA).

## RESULTS

### Participant flow and baseline characteristics

A total of 46 patients with frozen-phase adhesive capsulitis were enrolled and randomized into either the HA injection group (*n* = 21) or the rehabilitation group (*n* = 25). In the HA group, 1 participant was lost to follow-up by week 4, with 20 participants completing the final 26-week evaluation. In the rehabilitation group, 3 participants were lost to follow-up by week 4 and 2 additional participants by week 6; thus, a total of 20 participants completed the 26-week evaluation.

**Fig. 2:**
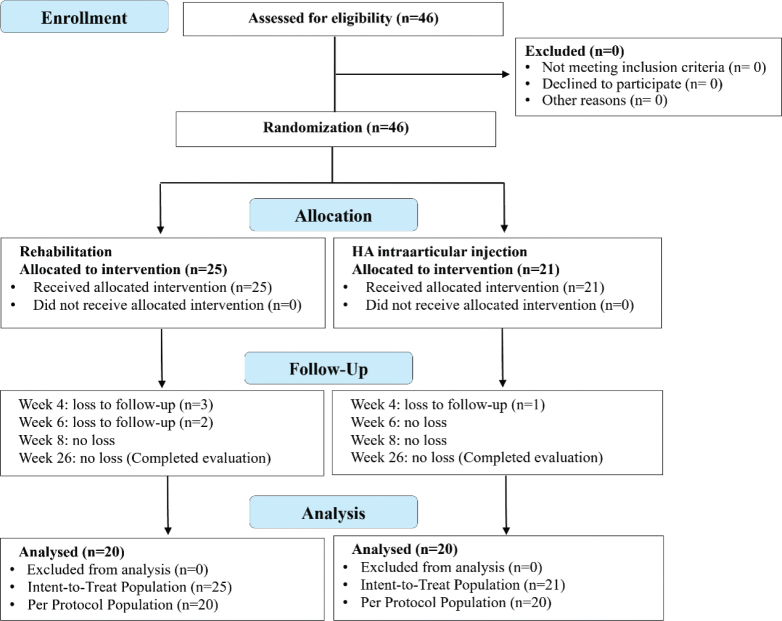
CONSORT flowchart of participant enrollment, allocation, follow-up, and analysis in both treatment groups.

Analysis was performed on both the intention-to-treat (*n* = 21 for HA, *n* = 25 for REH) and per-protocol (*n* = 20 for both groups) populations, as shown in Fig. 2. Overall, 1 participant in the HA group and 5 in the rehabilitation group were lost to follow-up, and no adverse events were reported in either group.

As summarized in Table I, baseline demographic and clinical characteristics, including age, sex, body mass index, comorbidities (diabetes mellitus and hypertension), and treatment side, were well balanced between groups, with no statistically significant differences observed (all *p* > 0.05). These findings confirm the adequacy of randomization and the comparability of groups at study entry.

### SPADI score outcomes

At baseline, SPADI total and subscale scores were comparable between groups, with no significant differences observed. Over the 26-week follow-up, both groups demonstrated statistically significant within-group improvements in SPADI pain, function, and total scores (*p* < 0.01 at all follow-up points). Between-group differences did not reach statistical significance at any time point, although numerical trends favored the HA group.

**Fig. 3:**
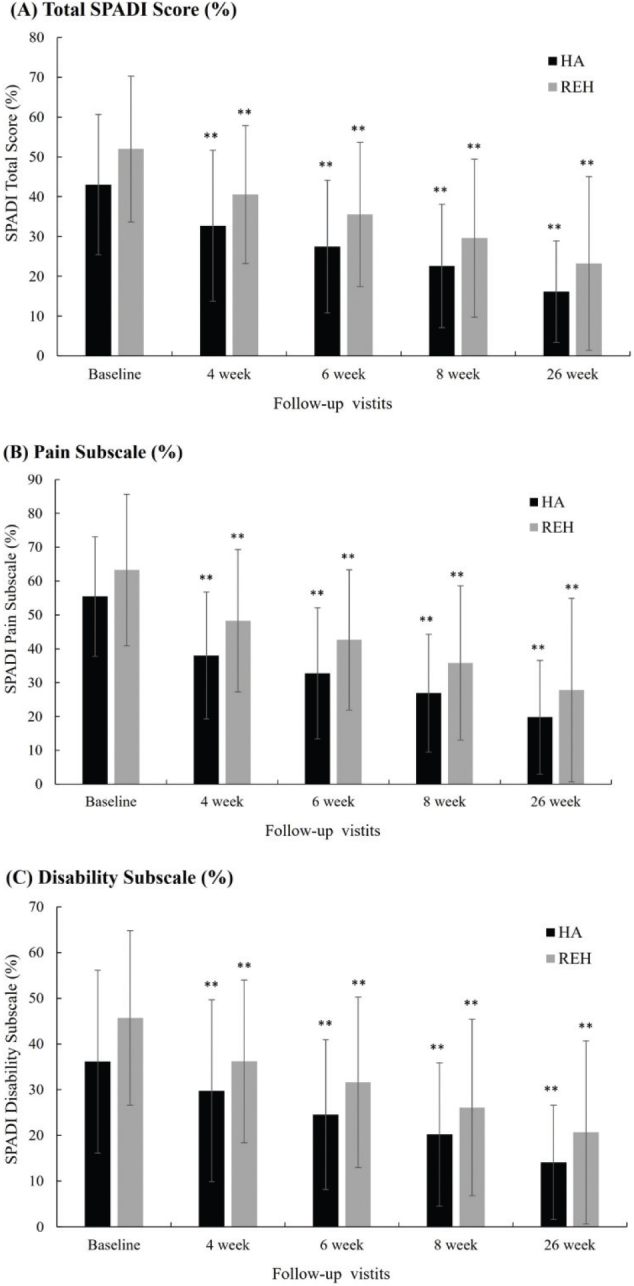
SPADI score changes in the HA injection (HA) and rehabilitation (REH) groups. (A) Total SPADI score; (B) Pain subscale; (C) Disability subscale across 26 weeks. Error bars represent standard deviation. *p<0.05, **p<0.01 compared with baseline within group.

As shown in Table II and Fig. 3, the HA group exhibited a reduction in total SPADI score from 43.03 ± 17.65 at baseline to 16.14 ± 12.77 at week 26, while the rehabilitation group improved from 51.97 ± 18.35 to 23.21 ± 21.83. Specifically, pain scores decreased by 64.3% in the HA group (from 55.43 ± 17.67 to 19.80 ± 16.77) and by 56.1% in the rehabilitation group (from 63.28 ± 22.40 to 27.80 ± 27.12). Functional limitation also improved, with a 60.9% reduction in the HA group (from 36.14 ± 20.00 to 14.12 ± 12.49) and a 54.8% reduction in the rehabilitation group (from 45.70 ± 19.07 to 20.67 ± 20.02).

**Table II:** Shoulder Pain and Disability Index (SPADI) scores at each follow-up visit within groups

Group	Baseline	Week 4	Week 6	Week 8	Week 26
Pain score (%)					
HA	55.43 ± 17.67	38 ± 18.76**	32.7 ± 19.36**	26.9 ± 17.38**	19.8 ± 16.77**
REH	63.28 ± 22.4	48.27 ± 21.01**	42.6 ± 20.76**	35.8 ± 22.77**	27.8 ± 27.12**
Function score (%)					
HA	36.14 ± 20	29.78 ± 19.92**	24.55 ± 16.42**	20.23 ± 15.65**	14.12 ± 12.49**
REH	45.7 ± 19.07	36.2 ± 17.81**	31.62 ± 18.66**	26.11 ± 19.3**	20.67 ± 20.02**
Total score (%)					
HA	43.03 ± 17.65	32.71 ± 18.98**	27.46 ± 16.68**	22.61 ± 15.49**	16.14 ± 12.77**
REH	51.97 ± 18.35	40.52 ± 17.34**	35.54 ± 18.12**	29.57 ± 19.89**	23.21 ± 21.83**

Values represent mean ± SD. * *p* < 0.05,

** *p* < 0.01 compared with baseline within the same group. Between-group comparisons at each time point showed no statistically significant differences (independent *t*-tests, *p* > 0.05). No comparisons were performed between follow-up time points. HA: hyaluronic acid injection group; REH: rehabilitation group.

Taken together, both interventions produced clinically meaningful improvements in pain and functional outcomes among patients with frozen-phase adhesive capsulitis, with comparable overall efficacy between groups.

### Range of motion outcomes

Baseline ROM values were numerically higher in the HA group for most parameters. However, between-group comparisons revealed no statistically significant differences at baseline except for active external rotation, which was significantly higher in the HA group (*p* = 0.042). Over the 26-week follow-up, both groups demonstrated significant within-group improvements across all measured parameters (*p* < 0.05 or *p* < 0.01). Between-group differences did not reach statistical significance at any time point. Although numerical differences were observed in certain ROM parameters, no consistent between-group advantage was demonstrated.

**Fig. 4:**
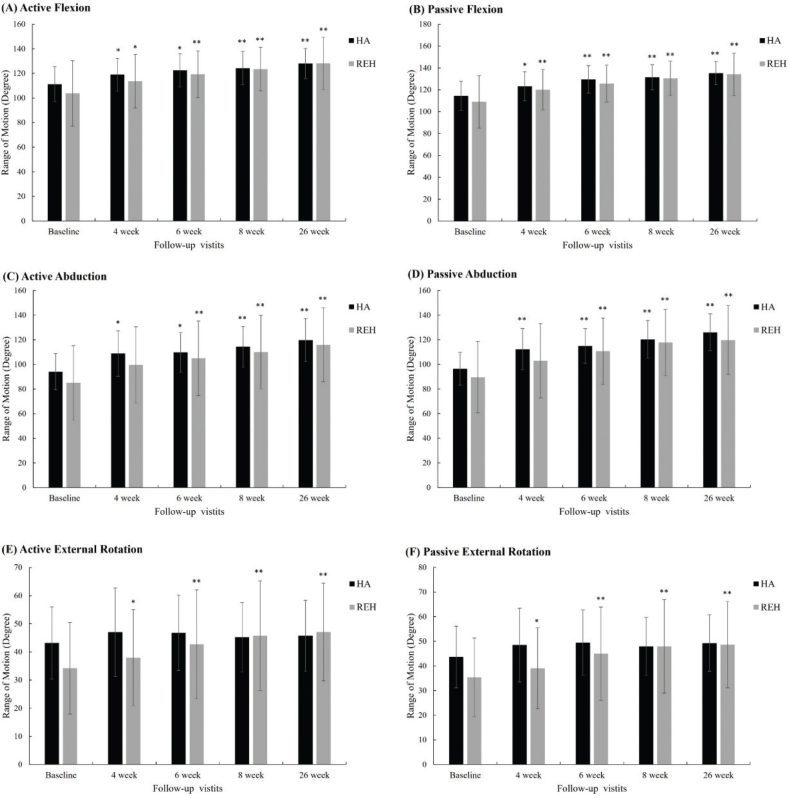
Shoulder range of motion (ROM) changes over time in the HA injection (HA) and rehabilitation (REH) groups. (A) Active Flexion, (B) Passive Flexion, (C) Active Abduction, (D) Passive Abduction, (E) Active External Rotation, (F) Passive External Rotation. Error bars represent standard deviation. *p<0.05, **p<0.01 compared with baseline within group.

As shown in Table III and Fig. 4, active flexion in the HA group improved from 111.19° ± 14.31 at baseline to 128.00° ± 12.29 at week 26, while the rehabilitation group improved from 103.80° ± 26.59 to 128.16° ± 21.23. Similar gains were observed in passive flexion, with increases from 114.52° ± 13.22 to 135.25° ± 10.45 in the HA group and from 109.00° ± 24.02 to 134.21° ± 19.38 in the rehabilitation group.

**Table III:** Active and passive shoulder range of motion (ROM) in degrees at each follow-up visit

Group	Baseline	Week 4	Week 6	Week 8	Week 26
Active Flexion					
HA	111.19 ± 14.31	119 ± 13.24*	122.5 ± 13.52*	124.25 ± 13.6**	128 ± 12.29**
REH	103.8 ± 26.59	113.64 ± 21.78*	119.25 ± 19.01**	123.5 ± 17.63**	128.16 ± 21.23**
Passive Flexion					
HA	114.52 ± 13.22	123.25 ± 13.21*	129.5 ± 12.66**	131.5 ± 11.48**	135.25 ± 10.45**
REH	109 ± 24.02	120 ± 18.64**	125.75 ± 16.88**	130.5 ± 15.64**	134.21 ± 19.38**
Active Abduction					
HA	94.05 ± 14.72	108.75 ± 18.56*	109.75 ± 16.26*	114.25 ± 16.57**	119.75 ± 17.51**
REH	85 ± 30.24	99.55 ± 30.86	105 ± 30.22**	110 ± 29.87**	115.79 ± 30.01**
Passive Abduction					
HA	96.43 ± 13.34	112.25 ± 16.82**	115 ± 13.95**	120.25 ± 15.34**	126 ± 14.92**
REH	89.6 ± 29.12	102.95 ± 30.18	110.75 ± 26.91**	117.75 ± 26.92**	119.74 ± 28.11**
Active External Rotation					
HA	43.19 ± 12.8	47 ± 15.76	46.75 ± 13.4	45.25 ± 12.3	45.75 ± 12.59
REH	34.2 ± 16.31	37.95 ± 17.09*	42.75 ± 19.3**	45.75 ± 19.49**	47.11 ± 17.35**
Passive External Rotation					
HA	43.67 ± 12.46	48.5 ± 14.96	49.5 ± 13.27	48 ± 11.74	49.25 ± 11.5
REH	35.4 ± 15.94	39.09 ± 16.45	45 ± 18.92**	48 ± 19.02**	48.68 ± 17.47**

Values are expressed as mean ± SD.

* *p* < 0.05,

** *p* < 0.01 compared with baseline within the same group. Groups were generally comparable at baseline, and no consistent between-group differences were observed during follow-up. No statistical comparisons were performed between follow-up time points. HA: HA injection group; REH: rehabilitation group.

In abduction, the HA group improved in active ROM from 94.05° ± 14.72 to 119.75° ± 17.51 and in passive ROM from 96.43° ± 13.34 to 126.00° ± 14.92, with numerically greater change scores observed in the rehabilitation group, although these differences did not reach statistical significance. For external rotation, both groups achieved significant within-group gains; the rehabilitation group showed greater increases in active ROM, whereas the HA group maintained higher passive ROM values throughout follow-up.

Taken together, these findings indicate that both ultrasound-guided HA injection and supervised rehabilitation produced meaningful and clinically relevant improvements in shoulder ROM during the frozen phase of adhesive capsulitis.

### Safety profile

No adverse events, complications, or treatment-related withdrawals were reported throughout the study. Both the ultrasound-guided intra-articular HA injection and the supervised rehabilitation program were well tolerated, confirming their safety in the management of frozen-phase adhesive capsulitis.

## DISCUSSION

This randomized controlled trial evaluated the efficacy of ultrasound-guided intra-articular hyaluronic acid (HA) injection compared with supervised physical rehabilitation in patients with frozen-phase adhesive capsulitis. Both interventions produced marked and clinically meaningful improvements in pain, function, and shoulder range of motion (ROM) over the 26-week follow-up. The primary endpoint – change in total SPADI score at week 26 – showed significant within-group improvement in both arms, with overall outcomes comparable between them. Although numerical differences were observed across certain ROM parameters, these variations did not demonstrate a consistent between-group advantage and should be interpreted as exploratory rather than indicative of treatment superiority.

Our findings are consistent with a 2022 meta-analysis by Mao et al. (12), which reported that intra-articular HA provides significant benefits in pain relief and ROM recovery, particularly when administered during the frozen stage of the disease. In our study, both treatment arms achieved statistically significant improvements in SPADI pain and function scores over time. Although improvements were observed as early as week 4 in the HA group, between-group differences were not statistically significant. Hence, any potential advantage in early symptom relief should be interpreted with caution.

Mechanistically, HA contributes to clinical improve-ment through its viscoelastic and anti-inflammatory properties. It enhances synovial lubrication, inhibits nociceptive signaling, and modulates fibrotic cytokine activity (20–22). Preclinical studies have shown that HA downregulates the expression of profibrotic markers such as transforming growth factor-beta (TGF-β) and interleukin-6 (IL-6) in capsular fibroblasts, thereby reducing capsular thickening and adhesion formation (18, 23). These biological effects are particularly relevant in the frozen phase of adhesive capsulitis, where capsular contracture is the principal barrier to mobility. In our study, both groups showed improvements in passive and active ROM across all measured domains. Although between-group differences did not reach statistical significance, numerical variations were observed across different ROM parameters. The HA group maintained slightly higher absolute ROM values in certain domains, whereas greater change scores were generally observed in the rehabilitation group across flexion, abduction, and external rotation. However, these numerical differences were inconsistent over time and did not demonstrate statistically significant between-group effects. Given that the study was powered based on the primary SPADI outcome rather than secondary ROM measures, future larger and adequately powered trials are warranted to further explore potential differences in specific mobility domains.

The HA group exhibited narrower standard deviations in some ROM measures, indicating less variability in response; however, the clinical relevance of this observation remains uncertain. Additionally, the HA group maintained higher absolute ROM values throughout follow-up, despite external rotation gains being greater in the rehabilitation group. These observations may reflect differences in therapeutic mechanisms between passive joint distension and structured stretching protocols, rather than a clear difference in overall efficacy. Notably, external rotation – often regarded as a hallmark limitation in adhesive capsulitis – improved in both groups, with greater gains observed in the rehabilitation arm. These findings imply distinct, potentially complementary mechanisms of action between passive joint distension and active stretching, warranting further study of combination approaches.

Although corticosteroid injections are widely used for short-term symptom relief in adhesive capsulitis, concerns remain regarding tendon weakening, chondral toxicity, and systemic effects, particularly with repeated use (10, 24). In contrast, HA offers a favorable safety profile, with no adverse events reported in our cohort – consistent with previous literature (14, 25). Moreover, HA is not associated with tissue degeneration and is well tolerated across age groups and comorbidity profiles, making it a valuable alternative for patients at risk of steroid-related complications.

Our results also align with those of Park et al. (18), who demonstrated in a prospective trial that ultrasound-guided capsular distension with HA led to significant clinical improvement compared with physical therapy alone. Similarly, Calis et al. found that intra-articular sodium hyaluronate provided comparable outcomes to corticosteroids in patients with adhesive capsulitis, without the associated risks (13).

### Strengths and limitations

This study has several strengths. It is one of the few randomized controlled trials directly comparing ultrasound-guided HA injection with structured rehabilitation in the frozen phase of adhesive capsulitis – a therapeutic window where evidence remains limited. The prospective randomized design, standardized treatment protocols, and high follow-up completion rate (87%) enhance the reliability of the findings. Importantly, both interventions produced clinically meaningful improvements in pain, function, and ROM, supporting their roles as effective non-surgical options. HA, in particular, demonstrated an excellent safety profile with no adverse events, underscoring its utility as a non-steroidal alternative for patients who may not be candidates for corticosteroid therapy.

Nonetheless, certain limitations should be acknowledged, including the modest sample size, lack of blinding, and absence of a sham control. In addition, baseline ROM values were numerically higher in the HA group across most parameters, with active external rotation demonstrating a statistically significant between-group difference at baseline (*p* = 0.042). This baseline difference may have contributed to variations in change scores, particularly for external rotation, and should therefore be considered when interpreting the secondary ROM findings.

An imbalance in loss to follow-up was observed between groups, with a higher attrition rate in the rehabilitation arm compared with the HA injection arm (5 vs 1 participants). This difference may have introduced potential attrition bias. One possible explanation is the greater time commitment required for supervised rehabilitation sessions compared with the shorter treatment course of HA injections. Importantly, both intention-to-treat and per-protocol analyses yielded comparable results, suggesting that the impact of differential attrition on the primary outcomes was likely limited. These factors should be considered when interpreting the findings. However, the consistency of improvements across multiple outcome measures and the comparable efficacy observed between groups suggest the robustness of the results. Future larger, multicenter trials are warranted to confirm these findings and to determine whether specific patient subgroups may derive differential benefit from either intervention.

### Conclusion

Both ultrasound-guided intra-articular hyaluronic acid injection and supervised physical rehabilitation resulted in significant within-group improvements in pain, function, and shoulder range of motion among patients with frozen-phase adhesive capsulitis. No statistically significant differences were observed between groups at any follow-up point. These findings indicate that both interventions represent effective and well-tolerated non-surgical options for managing adhesive capsulitis during the frozen stage. Further large-scale, multicenter studies are warranted to confirm these results and to identify potential patient subgroups who may derive greater benefit from each treatment.
